# Risk of Developing Major Mental Disorders in Hypertensive Patients: A Retrospective Observational Study Using the Common Data Model

**DOI:** 10.1192/j.eurpsy.2025.851

**Published:** 2025-08-26

**Authors:** H.-C. Kim

**Affiliations:** 1 Psychiatry and Brain Research Institute, Keimyung University School of Medicine, Daegu, Korea, Republic Of

## Abstract

**Introduction:**

Chronic medical conditions like hypertension may increase the risk of mental disorders such as anxiety and depression.

**Objectives:**

This study aimed to investigate if hypertension is associated with a higher incidence of major mental disorders, including anxiety disorders, depressive disorders, bipolar disorders, psychotic disorders, sleep disorders, vascular dementia, and Alzheimer’s dementia, compared to controls.

**Methods:**

I analyzed standardized data from patients with hypertension (n = 48,466) and those without hypertension (n = 442,660) at a university hospital. Clinical data was standardized into a common data model. Using propensity score matching (PSM) at a 1:5 ratio, I compared the incidence of mental disorders between the hypertension and control groups over a 5-year period. A multivariate Cox proportional hazards model was used to estimate the risk of mental disorders, with hazard ratios (HR) and 95% confidence intervals (CI).

**Results:**

After PSM, the hypertension group had a higher prevalence of being elderly (over 60 years old) and having conditions like diabetes, hyperlipidemia, atrial fibrillation, cerebrovascular disease, and heart disease compared to controls. The hypertension group also had significantly increased use of antithrombotic agents, beta-blockers, calcium channel blockers, diuretics, acid-related disorder drugs, diabetes medications, lipid-modifying agents, and opioids. The incidence rates per 1,000 patient-years for mental disorders were as follows: anxiety disorders (7.22 vs. 4.49), depressive disorders (8.51 vs. 5.47), bipolar disorders (1.13 vs. 0.91), psychotic disorders (0.18 vs. 0.22), sleep disorders (16.32 vs. 8.60), vascular dementia (0.77 vs. 0.14), and Alzheimer’s dementia (9.29 vs. 2.53). Compared to controls, the hypertension group had a higher risk of developing vascular dementia (HR, 6.03; 95% CI, 4.34–8.44; p<0.01), Alzheimer’s dementia (HR, 3.89; 95% CI, 3.56–4.24; p<0.01), sleep disorders (HR, 1.96; 95% CI, 1.85–2.07; p<0.01), anxiety disorders (HR, 1.69; 95% CI, 1.56–1.83; p<0.01), and depressive disorders (HR, 1.64; 95% CI, 1.52–1.76; p<0.01). There were no significant differences for bipolar disorders (HR, 1.17; 95% CI, 0.95–1.43; p=0.12) or psychotic disorders (HR, 0.85; 95% CI, 0.51–1.34; p=0.50).

**Conclusions:**

Hypertensive patients have an increased risk of major mental disorders, particularly vascular dementia, Alzheimer’s dementia, sleep disorders, anxiety disorders, and depressive disorders. Older age, age-related diseases, and various medication uses contribute to this increased risk.

**Disclosure of Interest:**

None Declared

